# Privacy-preserving framework for genomic computations via multi-key homomorphic encryption

**DOI:** 10.1093/bioinformatics/btae754

**Published:** 2025-01-31

**Authors:** Mina Namazi, Mohammadali Farahpoor, Erman Ayday, Fernando Pérez-González

**Affiliations:** Internet Interdisciplinary Institute (IN3), Open University of Catalonia, Barcelona 08018, Spain; Computer and Data Engineering School, Case Western Reserve University, Cleveland, OH 44106, United States; Department of Network Engineering, Universitat Politècnica de Catalunya, Barcelona 08034, Spain; Department of Electrical Engineering and Computer Science, Case Western Reserve University, Cleveland, OH 44106, United States; Signal Processing in Communication Group, School of Telecommunications Engineering, University of Vigo, Vigo 36310, Spain

## Abstract

**Motivation:**

The affordability of genome sequencing and the widespread availability of genomic data have opened up new medical possibilities. Nevertheless, they also raise significant concerns regarding privacy due to the sensitive information they encompass. These privacy implications act as barriers to medical research and data availability. Researchers have proposed privacy-preserving techniques to address this, with cryptography-based methods showing the most promise. However, existing cryptography-based designs lack (i) interoperability, (ii) scalability, (iii) a high degree of privacy (i.e. compromise one to have the other), or (iv) multiparty analyses support (as most existing schemes process genomic information of each party individually). Overcoming these limitations is essential to unlocking the full potential of genomic data while ensuring privacy and data utility. Further research and development are needed to advance privacy-preserving techniques in genomics, focusing on achieving interoperability and scalability, preserving data utility, and enabling secure multiparty computation.

**Results:**

This study aims to overcome the limitations of current cryptography-based techniques by employing a multi-key homomorphic encryption scheme. By utilizing this scheme, we have developed a comprehensive protocol capable of conducting diverse genomic analyses. Our protocol facilitates interoperability among individual genome processing and enables multiparty tests, analyses of genomic databases, and operations involving multiple databases. Consequently, our approach represents an innovative advancement in secure genomic data processing, offering enhanced protection and privacy measures.

**Availability and implementation:**

All associated code and documentation are available at https://github.com/farahpoor/smkhe.

## 1 Introduction

Substantial progress has been made in reducing the costs of DNA sequencing. Consequently, genomics research has paved the way for personalized medicine (also known as genomic medicine). Individuals use their genomes to learn about their health, origins, and genetic compatibilities with potential partners. This trend also caused the launch of health-related websites and direct-to-consumer (DTC) genomic service providers, in which individuals can share their genomic data [e.g. OpenSNP (https://opensnp.org) or 23andMe (https://www.23andme.com/en-int/)]. However, significant privacy risks emerge when sharing genomic data, which carries sensitive information, such as disease predisposition (e.g. for Alzheimer’s), ancestors, and physical attributes of its owner. The issue of genomic privacy is further compounded by the fact that an individual’s genome is inherently linked to the genomes of their family members, resulting in interconnected privacy risks. However, individuals need to provide access to their genomes for analyses by service providers to advance personalized medicine, facilitate medical research, and benefit from DTC services. The inherent conflict between the value of genomic data and its potential privacy implications emphasizes the necessity for privacy-preserving solutions to analyze such data effectively.

Existing privacy-preserving solutions for genomic data analyses can be categorized as cryptographic solutions (e.g. multiparty computations) and distortion-based solutions (e.g. the ones that are based on differential privacy (Hardt and Talwar 2010, Fienberg *et al.* 2011, Johnson and Shmatikov 2013, Yu *et al.* 2014). Differential privacy typically introduces excessive noise to the intermediate data, resulting in inaccurate models (Jayaraman and Evans 2019). Distributed solutions are limited to handling basic calculations and are unsuitable for complex tasks (Froelicher *et al.* 2017). Since our goal is to provide direct information processing to enable various operations on data from several data owners, we focus on cryptographic solutions. Among existing cryptographic solutions for the privacy-preserving analyses of genomic data, some of the most notable ones are based on homomorphic encryption (Kantarcioglu *et al.* 2008, Ayday *et al.* 2012, 2013a,b, Namazi *et al.* 2016, 2019). However, these solutions have certain drawbacks. First, they each attempt to solve a particular genomic data analyses problem using cryptographic-based schemes. Also, they mainly process each party’s genomic data separately, making processing multiple parties’ data simultaneously infeasible. Thus, their interoperability (i.e. their ability to work together in an extensive genomic data-sharing framework) and practicality are limited since using them together creates significant security and efficiency problems.

In our proposed framework, a multi-key homomorphic (mKH) encryption scheme (Chen *et al.* 2019) is utilized by a cloud-based server to ensure data privacy while performing analyses on patients’ encrypted data. Unlike previous techniques, our protocol requires minimal engagement from the parties involved and does not rely on a proxy for assistance. Additionally, each data owner can encrypt their genomic data using unique keys, mitigating the risk of a single point of failure. This approach eliminates the potential security breach of the entire protocol if one party’s key is compromised. By maintaining data encryption throughout the analyses process and enabling operations on combined records encrypted with different keys, our framework offers a natural protection against data breaches.

Our study aims to address these limitations by employing an mKH encryption scheme. We present a comprehensive protocol capable of conducting diverse genomic analyses on a moderate or small scale, facilitating interoperability among individual genome processing and enabling multiparty tests, analyses of genomic databases, and operations involving multiple databases. This approach offers unique advantages in key management and flexibility for multiparty computations while presenting new challenges in computational overhead and scalability.

It is crucial to note that while our framework provides a novel approach to privacy-preserving genomic data processing, it is not designed to replace systems optimized for large-scale genomic studies. Instead, it offers a complementary solution for scenarios involving individual records or medium-scale databases, Where the capacity to store encrypted data for multiple potential analyses is especially beneficial.

We summarize the main **contributions** of this as follows:

- We propose a privacy-preserving cloud-based framework supporting various analyses of individuals’ genomic data. It enables one-time encryption of genomic data and ensures interoperability between different privacy-preserving use cases.
- Our framework empowers genomic data owners to encrypt their information using unique public keys, eliminating the risk of a single point of failure. Additionally, it facilitates the analyses of combined genomic records encrypted with different keys, enhancing interoperability and preserving privacy.
- We evaluate our proposed framework’s run-time performance and scalability for various analyses. Our findings indicate that the framework’s run-time performance scales linearly with the database size. Moreover, it performs comparably to state-of-the-art techniques focusing on specific privacy-preserving genomic data analyses problems.
- Finally, we analyze the security of the proposed scheme and provide a formal proof.

The rest of the paper is organized as follows. We summarize the related work in Section 2. An overview of the building blocks and the core cryptosystem are provided in Section 3. We discuss the details of the proposed framework in Section 4, and its security analyses of the proposed framework are discussed in Section 5. We evaluate the practicality of the proposed framework in Section 6. In Section 7, we discuss potential extensions of the proposed framework. Finally, the conclusion is presented in Section 8.

## 2 Related work

Cryptographic solutions have been proposed for genomic privacy protection (Ayday *et al.* 2013a,b,c, 2019, Naveed *et al.* 2014, Namazi *et al.* 2016, 2019, Troncoso-Pastoriza *et al.* 2017). Within the realm of privacy-enhancing technologies for genomic data, an important category focuses on private clinical genomics, which directly affects individuals’ lives. This category encompasses various genetic testing applications such as susceptibility, paternity, personalized medicine, ancestry, compatibility, and identity testing. These tests can be conducted by processing genomic data stored on individuals’ devices or delegating the analyses to third parties, utilizing different cryptographic methods. This area of research is crucial for ensuring the privacy and security of sensitive genomic information while enabling valuable genomic testing applications.

Baldi *et al.* (2011) developed cryptographic techniques for privacy-preserving computations on genomic data using private set intersection cardinality (PSI-CA) protocol (Freedman *et al.* 2004). They suggested that individuals perform tests (such as personalized medicine) with no third-party contribution to ensure the trustworthiness and accuracy of DNA test results. Their method suggests that the parties are their own data centers and use their local devices. In contrast, our method avoids storing and processing genomic information on personal devices. Blanton and Bayatbabolghani (2016) suggested various privacy-preserving paternity testing methods that focus on the secure outsourcing of sequence comparisons. They used a server-aided two-party computation, a Boolean circuit, and zero-knowledge proofs. Individuals are responsible for running the test by engaging in oblivious transfers. Hence, the required interactions may cause inefficiencies in the protocol. Where Blanton *et al.*’s approach is specifically designed for paternity testing scenarios, our solution incorporates this capability within a broader framework that supports multiple types of genomic analyses under the same encryption scheme, demonstrating improved practicality and efficiency in real-world deployments while maintaining equivalent privacy guarantees.

Aggregation of genomic data by performing sequence comparison and matching have been deployed in genomic testing methods (Troncoso-Pastoriza *et al.* 2007, Jha *et al.* 2008, De Cristofaro *et al.* 2013). It includes running privacy-preserving queries between two databases (centralized or distributed) to solve problems such as record linkage. Karakasidis and Verykios (2011) proposed a secure blocking and edit distance calculation to find, link, and integrate records of the same individuals among multiple databases. Although this solution is efficient, our proposed framework considers linking databases across organizations by processing several encrypted databases in a cloud environment. Wang *et al.* (2015) and Asharov *et al.* (2018) proposed secure protocols to solve a similar patient problem (in which a physician who holds the genome of their patient attempts to find other individuals with “close” genomic data). However, the interactions between the parties and the high amount of precomputation decrease the efficiency of these protocols.

On the other hand, another research line focuses on privacy-preserving statistical computations and training machine learning models in decentralized platforms. Froelicher *et al.* (2017, 2020) proposed a combination of homomorphic encryption and zero-knowledge proof system inside interactive protocols, including differential privacy. They proposed a secure and verifiable data-sharing platform that computes statistics and trains the machine learning models on distributed datasets. Corrigan-Gibbs and Boneh (2017) proposed Prio. A privacy-preserving system for the collection of aggregate statistics using secret-shared, noninteractive proofs over the values of all clients (e.g. the most popular location). With higher costs and re-initialization of some protocols, these solutions might tackle our defined problem in this paper. However, Froelicher *et al.* (2020) deploy an encoding protocol for the input data, which is an application-specific operation. Therefore, these encodings should be regenerated when switching between applications. On the other hand, our solution directly evaluates the ciphertexts encrypted with different keys without requiring additional encoding, key-switching, or re-encrypting for each test. Moreover, instead of these solutions that work with elliptic curve assumptions and deploy (only) additive homomorphic operations, we leverage a lattice-based cryptosystem that allows for broader (direct) operations considering quantum attacks. Although our solution is centralized, it also avoids a single point of failure.

## 3 Background

We briefly introduce the basic building blocks and encryption schemes in our proposed privacy-preserving genomic computations (PPGCs) framework. We summarize the most frequent notations in this paper in Table 1.

**Table 1.** Used notations.

Calligraphic L	Set of participants in the protocols
Uppercase boldface A	Matrices
Lowercase boldface a	Vectors
a⊗b	Tensor product
x←D	samples x according to distribution D
$V=\{H_{1},\ldots ,H_{N}\}$	Set of the participants
$X_{id}^{E}$	Encrypted X under public key id

### 3.1 Genomic background

The human genome, consisting of ∼3 billion base pairs, is 99.9% identical between individuals. The remaining 0.1% accounts for genetic diversity and is crucial for genomic analyses. Two key types of genetic variations are particularly relevant to our framework:

#### 3.1.1 Single-nucleotide polymorphisms

Single base pair mutations occur approximately once every 300 nucleotides. Single-nucleotide polymorphisms (SNPs) are typically biallelic and are encoded as {0,1,2}, representing the number of minor alleles present. This encoding allows for efficient computational analyses while retaining essential genetic information. SNPs are vital in genome-wide association studies (GWAS) and personalized medicine applications as markers for traits, disease susceptibility, and drug responses.

#### 3.1.2 Short tandem repeats

Also known as microsatellites, short tandem repeats (STRs) are repetitive sequences of 2−6 base pairs. The human genome contains about 700 000 STR loci, with the number of repeats varying between individuals. STRs are highly polymorphic, making them valuable for forensic genetics and kinship analyses. An individual’s STR profile typically consists of 13−20 core loci, each represented by two alleles indicating the number of repeats.

SNPs and STRs contain sensitive information about an individual’s genetic predispositions, ancestry, and potential health risks. This sensitivity necessitates robust privacy-preserving mechanisms for genomic data analyses, especially in clinical or research settings where data sharing is crucial for advancing our understanding of human genetics and its role in health and disease.

Our privacy-preserving framework is designed to securely handle both SNP and STR data, enabling various genomic analyses while maintaining data confidentiality. By supporting operations on encrypted genomic data, we aim to facilitate critical genetic studies and personalized medicine applications without compromising individual privacy.

### 3.2 Cryptographic primitives

We describe the core cryptographic schemes and their hardness assumptions, on which we rely when developing the proposed PPGCs framework.

#### 3.2.1 Ring-learning with errors

This is a hardness problem that the most recent lattice-based cryptosystems are based on, and an efficient algebraic variation of learning with errors (LWE) introduced by Lyubaskevsky *et al.* Lyubashevsky10. For a security parameter λ, let $f(x)=x^{n}+1$, where n is an integer of power-of-two. Let an integer q≥2, and R=Z[x]/f(x). Then, $R_{q}$ is the residue ring of R modulo integer q. The ring-learning with error (RLWE) with the parameters (n,q,χ,ψ) indicates that any polynomial number of samples of the form $(a_{i},b_{i}=s\cdot a_{i}+e_{i})\in R_{q}^{2}$, where $a_{i}$ is uniformly random in $R_{q}$, s is chosen from the key distribution χ over $R_{q}$, and $e_{i}$ is drawn from the error distribution ψ over R, the $b_{i}$’s are computationally indistinguishable from uniformly random elements of $R_{q}$.

#### 3.2.2 Multi-key homomorphic encryption scheme with packed ciphertext

A mKH encryption scheme can evaluate an arithmetic circuit on encrypted ciphertexts under different keys. Chen *et al.* (2019) developed an efficient mKH encryption scheme based on the RLWE hardness problem with a packed ciphertext. The method allows homomorphic evaluation on multiple data packed into a single polynomial after parties encrypt them under their public keys. Regarding decryption, each party partially decrypts its data and broadcasts the result. The final decryption is obtained by joining all the partially decrypted values.

We briefly describe the main algorithms of this mKH scheme (mKHSetUp, mKHKeyGen, mKHEnc, mKHPartDec, mKHFinDec, mKHEval), as it follows.

$\mathrm{mKHSetUp}(1^{\lambda })\to pp_{\mathrm{mkh}}$ : on the input of the security parameter λ, the algorithm outputs $pp_{\mathrm{mkh}}$, which inputs to all the other algorithms of this scheme.

$\mathrm{mKHKeyGen}(pp_{\mathrm{mkh}})\to (sk_{id},pk_{id},ek_{id})$ : the algorithm inputs the public parameters and outputs a private key, a public key, and an evaluation key for each party id∈V.

mKHEnc(μ,pk,a)→ct : the algorithm on plaintext μ∈R outputs the ciphertext ct.

$\mathrm{mKHPartDec}(c_{id}^{*},sk_{id})\to p_{id}^{B}$ : each party id∈V receives the evaluated ciphertext, and decrypts it with the corresponding private key $sk_{id}$ and returns the partially decrypted ciphertext as $p_{id}^{B}$.

$\mathrm{mKHFinDec}({\left\{p_{id}^{B}\right\}}_{id\in V})\to \mu$ : the algorithm uses the partial decryptions as input and returns the message.

$\mathrm{mKHEval}(C,({\bar{ct}}_{1},\ldots ,{\bar{ct}}_{N}),{\left\{pk_{id}\right\}}_{id\in V},{\left\{ek_{id}\right\}}_{id\in V})\to ct^{*}$ on a circuit C∈C, a vector $\bar{ct}=\{ct_{1},\ldots ,ct_{N}\}$ is multi-key encryption of a plaintext μ with respect to the secret key of N different parties, and the corresponding set of public/evaluation keys runs the homomorphic operations as follows.

**Addition.** The algorithm inputs two ciphertexts ${\bar{ct}}_{1},{\bar{ct}}_{2}\in R_{q}^{N+1}$, returns a ciphertext $ct^{*}={\bar{ct}}_{1}+{\bar{ct}}_{2}\;(\text{mod}\;q)$.

**Multiplication.** The algorithm inputs two ciphertexts ${\bar{ct}}_{1},{\bar{ct}}_{2}\in R_{q}^{N+1}$ calculate a tensor product of two multi-key ciphertexts ${\bar{ct}}^{\prime }={\bar{ct}}_{1}⊗{\bar{ct}}_{2}\;(\text{mod}\;q)$. The resulting ciphertext includes nonlinear values of secret keys $s_{id_{1}}$, $s_{id_{2}}$ related to two different parties.

In a homomorphic encryption scheme, after a single multiplication between two ciphertexts, the noise grows to $O(B_{\chi }^{2}\cdot B_{\psi }\cdot n^{\left(3/2\right)})$, where $B_{\chi }$ and $B_{\psi }$ are bounds on the magnitude of samples from the key and error distributions respectively, and n is the dimension of the RLWE problem. The evaluated ciphertext also includes some errors. Chen *et al.* (2019) introduced a relinearization technique called modulo-raising that allows for a reduction of the ciphertext ${\bar{ct}}^{\prime }$ back into a regular ciphertext. Formally, $ct^{*}\leftarrow \mathrm{Relin}({\bar{ct}}^{\prime },{\left\{(pk_{id},ek_{id})\right\}}_{id\in V})$.

Regarding the noise growth problem, without going into the details of noise estimation, the modulus-raising technique allows for noise reduction after multiplication, bringing it back to $O(B_{\chi }\cdot B_{\psi }\cdot n^{\left(1/2\right)}\cdot N)$, where N is the number of parties involved in the computation. Evaluating a degree of 2 polynomial would be sufficient due to the choice of our proposed framework. This marks a substantial advancement over naive approaches, ensuring noise growth remains linear in N rather than becoming exponential.

**Security.** The mKH encryption scheme with the packed ciphertext of Chen *et al.* (2019) is IND-CPA for semihonest adversaries with the above definition under the RLWE hardness assumption and the parameters (n,q,χ,ψ).

## 4 Proposed framework for PPGCs

Our proposed framework, described in this section, is the first to outsource encrypted genomic data storage to a cloud environment and supports various tests. In this framework, individuals’ genomic information is sequenced and stored confidentially in a cloud-based server. The cloud server processes different parts of the stored encrypted data for different genomic tests and functionalities.

### 4.1 System model and settings

The participants of the protocol include (i) a certified institution (CI) that sequences the biological samples of the parties, (ii) a key authority (KA) that generates public parameters of the scheme, (iii) a cloud-based server that behaves as a storage and processing unit (SPU) and, (iv) various types of data owners and queriers ($H_{1},\ldots ,H_{N}$, where N is the maximum number of the involved parties) for each application of the proposed framework.

**Figure 1.**
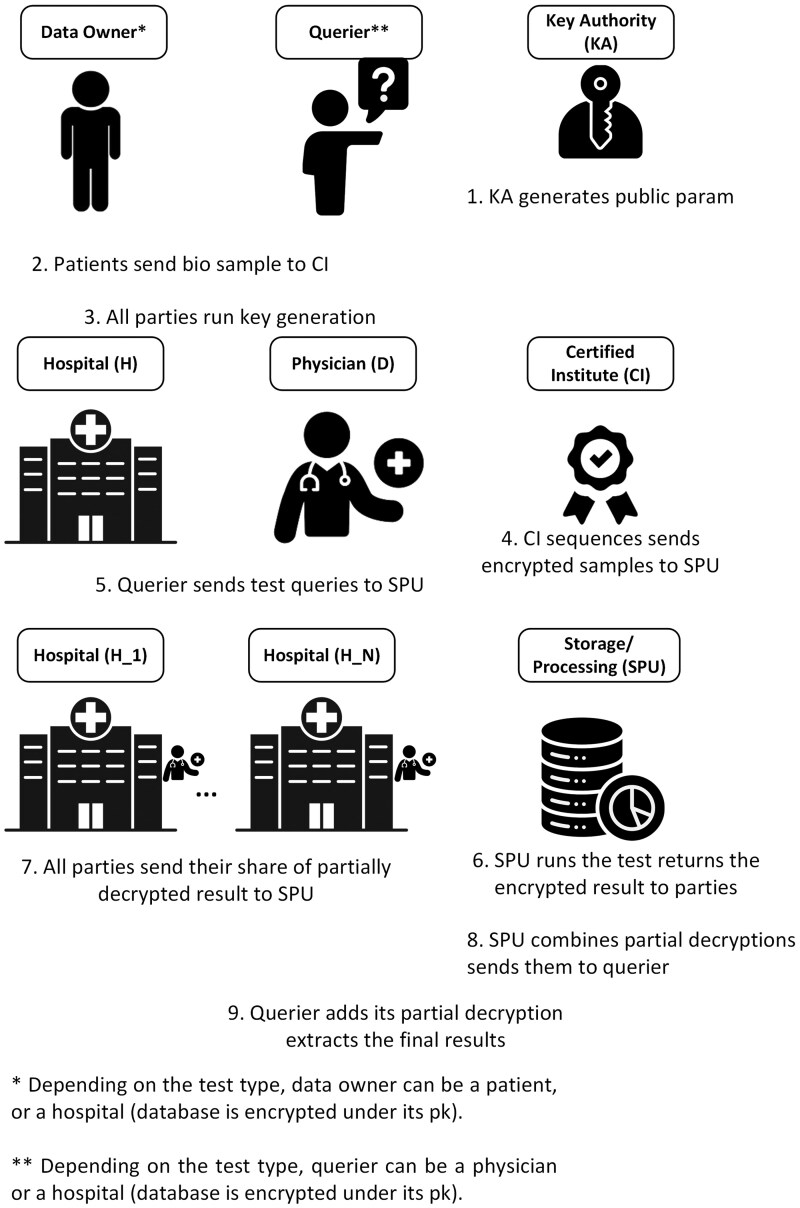
Proposed framework setting.

We assume each party generates its own private/public/evaluation key (the public and evaluation key pairs are public). The CI (or each party itself via an interface) encrypts the sequenced genomic data of the individuals under the public key of each party right after the sequencing. The SPU stores the encrypted genomic data and performs the required analyses on the individuals’ stored genomic data. It also provides the final result of a test to the corresponding parties. We show the general overview of our proposed framework setting in Fig. 1.

We develop the proposed framework assuming that the individuals’ genomes are stored at a cloud service provider (rather than storing the data locally at each data owner). This design choice is realistic, considering a computationally rich cloud’s higher computation power, data availability, and security. As the cryptographic building block, we utilize the mKH encryption scheme of Chen *et al.* (2019). This cryptosystem enables operations over data that is encrypted under different keys. Each user (data owner) encrypts their data under their public keys.

For decryption, each user partially decrypts the encrypted result and exchanges it with the SPU (or with the other parties if their interactions are permitted in the protocol). The final decryption is done by joining the partially decrypted values and the final party’s decrypted value. The requirement of such partial decryption inherently provides access control to the proposed framework.

Our framework enables various homomorphic operations on encrypted genomic data from multiple sources. These operations include addition, subtraction, comparison, and multiplication. We demonstrate our framework’s core operations through four key scenarios in genetic testing. We are excluding GWAS and large-scale data analyses from these operations and leaving them for future work.

- Individual genomic tests (e.g. Personalized Medicine): Calculates an individual’s genetic risk score for a particular condition based on their SNP profile.
  (1) $$S_{\text{PM}}=\left(\frac{1}{\Sigma_{j=1}^{n}c_{x,j}}\right)\cdot \Sigma_{j=1}^{n}\left(c_{x,j}\cdot f({\text{SNP}}_{P,j},{\text{pr}}_{x,j})\right)$$
  Where $c_{x,j}$ is the contribution of the jth SNP, ${\text{SNP}}_{P,j}$ is the jth SNP of patient, $pr_{x,j}$ is the probability associated with the jth SNP for disease x, and $f({\text{SNP}}_{P,j},{\text{pr}}_{x,j})={\text{pr}}_{x,j}^{{\text{SNP}}_{P,j}}\cdot {\left(1-{\text{pr}}_{x,j}\right)}^{1-{\text{SNP}}_{P,j}}.$ requires n multiplications and n−1 additions for weighted risk calculation across n SNPs, where n is the number of relevant SNPs.
- Multiparty tests (e.g. Paternity Testing): Compares genetic markers between a child and an alleged father to determine paternity. The test
  (2) $$S_{\text{PT}}=\left(\frac{1}{K}\right)\cdot \Sigma_{i=1}^{K}f(x_{i,1},x_{i,2},x_{i,1}^{\prime },x_{i,2}^{\prime })$$
  Where, K is the number of STR markers, $x_{i,1}$ and $x_{i,2}$ are the child’s STR values, and $x_{i,1}^{\prime }$ and $x_{i,2}^{\prime }$ are the alleged father’s STR values, and $f(x_{i},1,x_{i},2,x_{i}^{\prime },1,x_{i}^{\prime },2)=(x_{i},1-x_{i}^{\prime },1)(x_{i},1-x_{i}^{\prime },2)(x_{i},2-x_{i}^{\prime },1)(x_{i},2-x_{i}^{\prime },2)$. It involves 4K subtractions and 3K multiplications to compare K STR markers.
- Genomic database analyses (e.g. Similar Patient Search): Finds genetically similar individuals within a database, useful for identifying related conditions or treatments.
  (3) $$S_{\text{SP}}=\left(\frac{1}{L}\right)\cdot \Sigma_{i=1}^{L}f(a_{i},b_{i}).$$
  Where L is the length of the genetic profile, $a_{i}$ and $b_{i}$ are the i-th elements of profiles a and b, $f(a_{i},b_{i})=\text{min}(|a_{i}-b_{i}|,c)$, and c is a constant representing the maximum allowed difference. The test requires L subtractions and comparisons per patient pair.
- Multi-database operations (e.g. Record Linkage): Identifies and links records belonging to the same individual across different databases. It deploys Levenshtein (1966):
  (4) $$S_{\text{RL}}=\left(\frac{1}{\max(m,n)}\right)\cdot \Sigma_{i=1}^{\max(m,n)}f(a_{i},b_{i}).$$
  Where a and b are records of length m and n respectively, $f(a_{i},b_{i})=lev_{a,b}(i,j)$ is the distance between the first i characters of a and the first j characters of b, and $\left[a_{i}\neq b_{j}\right]$ is 0 when $a_{i}=b_{j}$, and 1 otherwise. It requires mn comparisons, additions, and min operations per record pair.

### 4.2 Threat model

We prove the security of our proposed scheme for semihonest adversaries based on the semantic security of the underlying mKH encryption scheme.

Consistent with previous works, the CI is assumed to be trusted since it has direct access to biological samples of the individuals for sequencing. The key authority KA, the SPU, data owners, and queriers (e.g. individuals or physicians who query for analyses) are semihonest and follow the protocols. They are not allowed to modify their inputs to obtain unauthorized information. However, they might be curious to obtain more information from their observed transactions.

Semihonest users (data owner or querier) might collude with the SPU. Since they follow the protocol instructions, the extracted results do not transmit helpful information to break the scheme’s security. The proposed framework requires partial decryption of other data owners to recover data or test results that belong to them. Such partial decryptions also provide internal access control to the proposed framework. We provide formal security definitions of the proposed framework for semihonest adversaries and the corresponding formal security proofs in Section 5.

While our security model provides strong guarantees against semihonest adversaries, it does not address potential inference attacks based on the results of multiple queries. Such considerations are beyond the scope of this work and represent an important area for future research.

### 4.3 Protocol overview

First, the key authority KA generates the public parameters. Each party generates and publishes its public/evaluation keys using these public parameters. Each party sends its biological sample for sequencing to the CI. The CI sequences these samples. The CI (or users) encrypts each genomic profile using each party’s public key and sends these encrypted profiles to the SPU for storage.

Without loss of generality, we assume that the operations (tests) at the SPU include data from N parties from the set $V=\{H_{1},\ldots ,H_{N}\}$. Any party $H_{i}\in V$ who starts the protocol is supposed to get the test result. However, this setting can be extended to newly joined parties. The SPU first chooses the particular SNP (or STR) profiles relevant to the required test. The SPU then runs the test on participants’ encrypted genomes by calling the evaluation algorithm of the mKH scheme (as described in Section 3.2.2). The computation result is encrypted under the public keys of all N participants.

Next, each test participant, except the $H_{i}\in V$, partially decrypts the encrypted result using its corresponding private key. Each participant returns their partially decrypted share to the SPU. Then, the SPU sends all partially decrypted shares to party $H_{i}$. Finally, $H_{i}$ runs the decryption algorithm over all the partially decrypted ciphertexts and recovers the test result.

The proposed framework’s computational analysis phase requires no interaction from data owners. However, the final decryption involves the parties’ participation due to the inherent security design of mKH encryption. Each party contributes their partial decryption to reconstruct the final result. The partial decryption step, though interactive, is minimal compared to traditional protocols that require continuous interaction during computation. Our framework reduces all participant interaction into this final phase, significantly reducing the overall communication overhead while maintaining the security guarantees of the mKH encryption scheme.

Moreover, it can support a *dynamic* nature. Public parameters published by the key authority enable the newly joined parties to generate their keys, encrypt their data, and store it at the SPU. Later, these parties can ask for analyses on their stored data at the SPU. Since genomic data are categorized as sensitive data based on GDPR, an ideal protocol (that stores and processes genomic data) should allow people to withdraw their consent to use their data. Deleting the parties and their information from the SPU is straightforward in the proposed scheme.

### 4.4 Privacy-preserving framework for genomic computations scheme in detail

In this section, we provide the details of our general framework for PPGC by describing the operations and interactions between the parties. In the following, we describe the key steps of the proposed framework.

$G_{H_{i}}$ represents the sequenced genomic data of each party $H_{i}\in V=\{H_{1},\ldots ,H_{N}\}$. The $G_{H_{i}}$ can be the SNP${}_{H_{i}}$ or the STR${}_{H_{i}}$ profile, depending on the application.

**Setup and key generation.** The KA runs mKHSetUp to generate $pp_{\mathrm{mkh}}$, which is then used as part of the input in all of the following algorithms. Then, each party $H_{i}$ runs $\mathrm{mKHKeyGen}(pp_{\mathrm{mkh}})\to (sk_{H_{i}},pk_{H_{i}},ek_{H_{i}})$. It outputs $\left(pp=pp_{\mathrm{mkh}},k_{H_{i}}=(sk_{H_{i}},pk_{H_{i}},ek_{H_{i}})\right)$, for each party $H_{i}\in V$. We also show this process in Algorithm 1.

Algorithm 1. Setup and key generation.

**Input:** Set of N individuals $V=\{H_{1},\ldots ,H_{N}$}.

**Output:**  $\left(pp,k_{H_{i}}\right)$ for each party $H_{i}\in V$. KA runs $\mathrm{mKHSetup}(1^{\lambda })\to pp_{\mathrm{mkh}}$;

Each $H_{i}$  **forall**  $H_{i}\in V$  **do** 
 

$$\left|\mathrm{mKHKeyGen}(pp_{\mathrm{mkh}})\to k_{H_{i}}=(sk_{H_{i}},pk_{H_{i}},ek_{H_{i}})\right.;$$

**end**

**Sequencing and encryption.** Each party $H_{i}$, for $H_{i}\in V$, sends its biological sample for sequencing to the CI who sequences them by running $\mathrm{Seq}({\text{DNA}}_{H_{i}})\to G_{H_{i}}$. The CI (or the parties) encrypts the genetic profiles of the individuals under each party’s public keys (or under the public key of the parties who have the data owners’ consent) $pk_{H_{i}}$ and sends these encrypted profiles $G_{H_{i}}^{E}$ to the SPU. We refer to Algorithm 2 for details.

Algorithm 2. Sequencing and encryption.

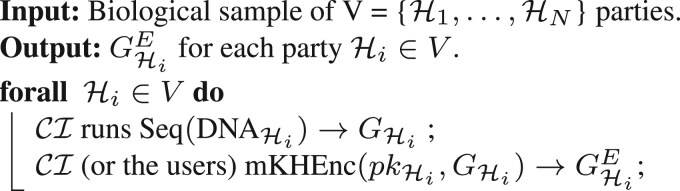

**Encrypted genomic test.** We assume the operation (test) at the SPU includes data from N parties, and party $H_{i}\in V$ is supposed to get the result of the test. After the test query, the SPU identifies the locations (on the genome) for each particular analyses and runs the test on patients’ encrypted genetic profiles with the public key of each patient. It calls the evaluation algorithm (mKHEval) of the multi-key encryption scheme (as discussed in Section 3.2.2) based on the required operations for different test types. The SPU releases the result of the queried test that is encrypted under the public keys of all N parties, $Z_{H_{i}}^{*}$. Algorithm 3 shows these interactions.

Algorithm 3. Encrypted genomic test.

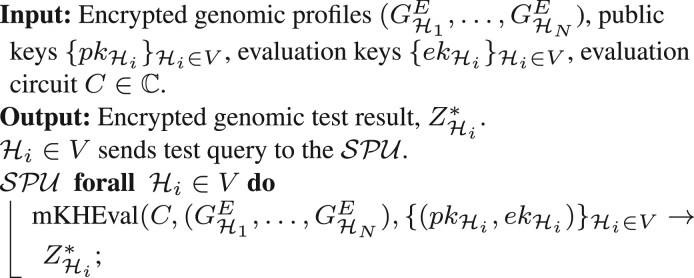

**Decryption of the test result.** Each party $H_{j}\in V$, with the exception of $H_{i}$, partially decrypts the jth entry of $Z_{H_{i}}^{*}$ by calling mKHPartDec using their private key $sk_{H_{j}}$ to obtain the partially decrypted test result $Z_{H_{i}}^{B}$and returns their partially decrypted share to the SPU. The SPU sends each $Z_{H_{i}}^{B}$ to party $H_{i}$. The $H_{i}$ runs the final decryption algorithm mKHFinDec over its own partially decrypted value, and all the received partially decrypted ciphertexts with $sk_{H_{i}}$ to recover the final test result, Z, in plaintext. Algorithm 4 shows the steps of this process.

Algorithm 4. Decryption of the test result.

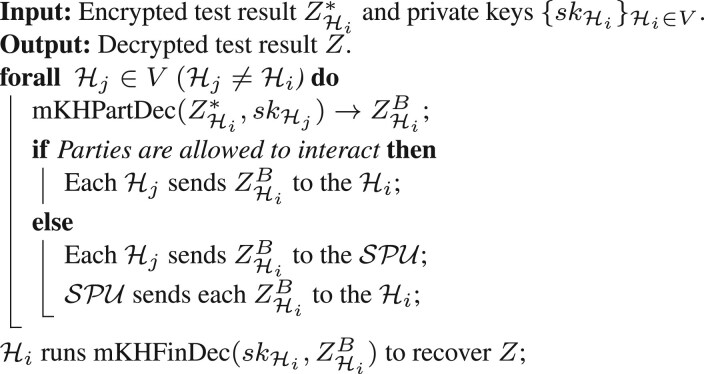

## 5 Security analyses

This section provides general security proof of our proposed privacy-preserving genomic data processing (PPGC) scheme.

Suppose there exists a probabilistic polynomial time (PPT) adversary A who breaks the security of the PPGC. Then, we can construct a PPT challenger B to break the semantic security of the underlying mKH scheme against semihonest adversaries.

Theorem 1. *The privacy-preserving interoperable genomic testing framework (PPGC) is semantically secure against PPT semi-honest adversaries if the underlying mKH encryption scheme is semantically secure.*

**Proof.**

Let the framework involve N parties. The following game describes the interaction between A (controlling SPU) and a challenger B (simulating the parties) and proceeds as:

**Setup:**  B is given the security parameter and public parameters $pp_{\mathrm{mkh}}$. It generates N key pairs and sends public and evaluation keys along with the public parameters to adversary A and keeps the private keys secret.

**Query:** Adversary A chooses an index k∈[N] and outputs a pair of message vectors $m_{1}={\text{DNA}}_{H_{l}}$, and $m_{2}={\text{DNA}}_{H_{j}}$ for $H_{l},H_{j}\in V$, and asks for running the sequencing and encryption algorithm.

**Challenge:** The challenger B chooses a random b←{0,1}, and returns the encrypted genomic sequences $G_{H_{b}}^{E}$ to B.

**Guess:** The adversary A can make polynomial-many mKHEval queries. Finally, A outputs a bit $b^{\prime }$. If $b^{\prime }=b$, then A wins.

 By assumption, A wins this game with a nonnegligible probability.

 B interacts with its own challenger C as:

**Setup**: The challenger C runs mKHSetup to obtain a challenge key pair and pass them to B. The latter runs $\mathrm{mKHKeyGen}(pp_{\mathrm{mkh}})\to k_{H_{i}}^{\prime }=(sk_{H_{i}}^{\prime },pk_{H_{i}}^{\prime }\;,ek_{H_{i}}^{\prime }{)}_{i\in [N]}$ and sends $pk_{H_{i}}^{\prime }$ with the public parameters to adversary A.

**Reduction:** When A sends genome challenge $m_{1}={\text{DNA}}_{H_{l}}$, and $m_{2}={\text{DNA}}_{H_{j}}$ for $H_{l},H_{j}\in V$, to B, the latter runs Seq and sends the genomic sequences to C. The challenger C runs mKHEnc, and returns the encrypted genomic sequences $G_{H_{b}}^{*E}$ to B.

**Challenge:**  B generates the N−1 key pairs following the exact steps of the algorithm. It embeds $G_{H_{b}}^{*E}$ as one of the N ciphertexts that A receives.

**Output:** When A outputs guess $b^{\prime }$, B outputs the same guess $b^{\prime }$ to C.

Since A wins the security game PPGC with a nonnegligible advantage, it can distinguish the ciphertext $G_{H_{b}}^{*E}$ corresponding to $m_{1}$ from $m_{2}$ under the public key $pk_{H_{i}}^{\prime }$. Thus, adversary B can leverage A to break the semantic security of the mKH scheme with a nonnegligible advantage.

Since we deploy a semantically secure mKH (as proved in Chen *et al.* 2019), which provides semantic security for the encryption based on the RLWE assumption, the advantage of B is negligible to output the correct bit; hence A’s. Therefore, our PPGC scheme is secure if the underlying mKH encryption scheme is semantically secure.

The security proofs of each application of Section 4.4 rely on this general security model. However, each application’s security definitions may differ since the participating parties’ roles can change depending on the application scenario. Note that different applications’ security goals are not conflicting; a party can be the data owner in one application and the querier in another. When a party is the data owner, its security goal is data privacy (or database). When the same party is a querier, its security goal is its data and the query’s result.

## 6 Implementation and evaluation

This section presents the implementation details of our privacy-preserving framework proposed in Section 4.4 and evaluates its performance across the four key genomic test scenarios introduced in Section 4.1. We analyze the computational complexity, provide runtime measurements, and discuss the scalability of our approach.

The complexity of parameter generation by the Key Authority KA is O(λ), where λ is the security parameter. Key generation for each party has a O(n log q) complexity, where n is the polynomial modulus degree, and q is the ciphertext modulus. These operations are performed once during the setup phase. The encryption process, carried out by the CI, has a O(n log q) complexity per genetic marker. However, the mKH multiplication is the proposed framework’s most computationally heavy operation. This operation, performed by the SPU, has a complexity of $C_{\mathrm{mult}}=O(N^{2}n\;\text{log}\;q)$, where N is the number of parties involved in the computation. Decryption takes O(1) complexity to decrypt one. We summarize the complexity analyses in Table 2.

**Table 2.** Complexity analyses of the Proposed PPIG Protocol (4), for security parameter λ, ring dimension *n*, and *N* parties.

**@** H	**@** CI	**@** SPU	**@** KA
O(1)	O(n log q)	$O(N^{2}n\;\text{log}\;q)$	O(λ)

The scalability of our framework depends on the computational cost of homomorphic operations and the noise growth in ciphertexts.

Homomorphic multiplication, which dominates the computational cost, has a complexity of $C_{\mathrm{mult}}$ that quadratically grows with the factor N, explaining the significant increase in runtime for multiparty and multi-database operations.

Regarding noise growth, our suggested choice of parameters allows for ∼102 multiplications with a degree-2 polynomial evaluation before decryption becomes unreliable.

Compared to traditional nonprivacy-preserving methods, which usually have linear complexity based on input size, our framework adds extra computational overhead to guarantee privacy. This overhead is manageable for individual-level and small-scale database operations. However, large-scale operations become more challenging, particularly those involving multiple databases or numerous parties.

We implemented our framework using the SMKHE library (https://github.com/farahpoor/smkhe), which provides mKH encryption capabilities for Chen *et al.*’s (2019) work. Our experiments were conducted on a Ubuntu Linux system on an Intel Core i7 of the 4-th Generation CPU, 16 GB RAM, and a 128 GB SSD.

We chose parameters to achieve 128-bit security based on the homomorphic encryption security standard (HEStd). We set the polynomial modulus degree $n=2^{14}$, with an appropriate coefficient modulus q and a plaintext modulus $t=2^{16}$. These parameters allow for a multiplicative depth of 2, sufficient for our genomic testing scenarios.

The analyses emphasizes that while our framework is efficient for individual and small-scale tests, it becomes challenging for large-scale database operations. Future optimizations could focus on reducing the number of homomorphic multiplications, running the program in parallel, or exploring hybrid approaches that combine homomorphic encryption with other privacy-preserving techniques for improved scalability in large-scale scenarios.

We assume that the setup phase is run once the protocol is initiated. The parameters are distributed to all the parties and phases; each party generates its keys, and the CI is the party that encrypts the data; the running time of running one mKH multiplication takes 13 033 ms, and the addition requires 3374 ms. With the input size of 13 (considering the STR markers in the paternity test), we obtain the results in 13 087 and 3381 ms. The running time slightly increases to 13 100 and 3408 ms for multiplication and addition if we run the experiment for a data size of 1000. When we introduce an input size of 8000, producing the test results takes 13 107 and 3417 ms. We observe that increasing the input size does not significantly impact the runtime for operations since the underlying encryption scheme operates on batches of data. The smaller input sizes (such as 10, 100, or even 1000) may fit into a single batch, leading to similar runtime results. Furthermore, the operations benefit from parallel processing, where computations are spread across multiple threads or vectorized, reducing the impact of increasing input sizes on runtime. We summarize these results in Table 3.

**Table 3.** Scalability analyses in the PPGC protocol in Section 4.

Input size/operation	mk. Mult.	mk. Add.
1	13 033 ms	3374 ms
13	13 087 ms	3381 ms
1000	13 100 ms	3408 ms
8000	13 107 ms	3417 ms

For larger input sizes beyond 8000, errors occur due to exceeding the available levels in the encryption scheme, which restricts the number of possible operations on the encrypted data. Homomorphic multiplication introduces higher runtime costs, mainly due to the need for relinearization and rescaling. The fixed computational cost of these homomorphic operations, combined with the constraints of the encryption scheme, leads to the observation that increasing input size only dramatically affects runtime once reaching the limits of the scheme.

Applying the results to various test types introduced in Section 4.4, with the maximum possible input size, the proposed scheme’s application to personalized medicine test 1 shows that the proposed protocol efficiently runs this formula through 2 homomorphic multiplication and 1 addition of the SNPs in 29 631 ms. Paternity testing 2 requires evaluating a multi-variant polynomial of degree 2 in $x_{i,1}$, which comprises four subtractions (implemented as additions) and three multiplications. The time that it takes to return the results is 52 989 ms. Our proposed scheme outputs the result of finding similar patient defined 3, through 5 mKH additions in 17 085, and it outputs the result of record linkage test with 10 additions in 34 170 ms. The summary is represented in Table 4.

**Table 4.** Run-time analyses for genetic test types 2, 1, 3, and 4, in Section 4.4, with input size 8000, based on their required number of operations.

Test type	Time
Individual records	29 631 ms
Multiple parties’ records	52 989 ms
A database analyses	17 085 ms
Multiple databases analyses	34 170 ms

We compare the proposed protocol’s performance with the state-of-the-art protocols such as the proposals represented in Asharov *et al.* (2018), Blanton and Bayatbabolghani (2016), Baldi *et al.* (2011), and Karakasidis and Verykios (2011). The evaluation results demonstrate varying performance characteristics across different genomic test scenarios. For individual genomic tests, our runtime rounded to 30 s compared to Baldi *et al.*’s 6.5 s reflects the additional computational overhead of mKH encryption. Similarly, our framework requires ∼53 s for multiparty tests versus Blanton *et al.*’s 45 s. At the same time, database analyses show our 17 s against Asharov *et al.*’s 15 s, and multi-database operations take 35 s compared to Karakasidis *et al.*’s 28 s. These differences in runtime performance derive from our framework’s comprehensive privacy-preserving approach using mKH encryption, which necessarily introduces additional computational complexity compared to more specialized solutions like PSI or garbled circuits used in existing works. In Table 5, we compare the running time of the proposed scheme for a fixed input with state-of-the-art articles and argue the advantages and disadvantages of deploying the proposed scheme.

**Table 5.** Performance comparison of PIPG with the running-time of schemes in Asharov *et al.* (2018), Blanton and Bayatbabolghani (2016), Baldi *et al.* (2011), Karakasidis and Verykios (2011) in seconds.

Test types/schemes	Our scheme	Other schemes
Individual records	30	Baldi *et al.* (2011): 6.5
Multiple parties’ records	53	Blanton and Bayatbabolghani (2016): 45
A database analyses	35	Karakasidis and Verykios (2011): 28
Multiple databases analyses	17	Asharov *et al.* (2018): 15

Our framework justifies its performance overhead with significant advantages. It eliminates precomputation phases and interactive rounds, provides enhanced privacy guarantees through homomorphic encryption, and supports multiple operations on the same encrypted data. Additionally, it can incorporate new types of genomic tests without system redesign, representing a significant advancement in PPGC despite a moderate increase in computational overhead.

## 7 Discussion

This section briefly discusses the proposed protocol’s security and privacy performance and our design choice.

### 7.1 Security/privacy guarantees

Our security evaluation shows the guarantees of the proposed scheme under the semihonest adversary model.

Because the SPU runs the evaluation algorithms over the parties’ encrypted genomic data, the input data remain confidential while performing the test. The parties who query the genomic test are the only authorized parties who can combine these partially decrypted parts and extract the plaintext result.

Some queries’ results leak information about the individuals’ genomic data (e.g. the size of the data or required operations). Inference (or a data owner’s sensitive information) from the query result requires additional privacy definitions and constructing a leakage function and its analyses. It is not considered here, as we only focus on privacy during the data processing. For instance, a physician may try to infer a patient’s SNPs from the result of a personalized medicine test or the size of the encrypted data set. We plan to consider such attacks in future work.

Our framework offers several unique advantages for specific genomic data processing scenarios. It allows data to be encrypted once and used for multiple analyses without re-encryption, providing a balance of security and flexibility not found in many existing systems. The multi-key approach eliminates single points of failure in key management, enhancing overall system security. However, it is worth acknowledging our approach’s limitations. The framework is not designed for complex, large-scale genomic studies. With our current implementation, such applications would face significant computational and storage overhead. Instead, our system is optimized for scenarios involving individual records or small-scale databases, where the ability to leave encrypted data for multiple potential analyses outweighs the need for complex statistical computations.

### 7.2 Design choice

We claim that personal devices like smartphones and laptops are vulnerable and not computationally powerful. Thus, they are not convenient to store sensitive information such as genomic data or the individuals’ medical records. Hence, (i) outsourcing the storage and processing of genomic data to a cloud server in a privacy-preserving way is a desired feature for individuals and genomic databases; (ii) achieving and developing minimal interactive schemes is a crucial requirement for the user-friendliness of a protocol. In the proposed framework, the parties (e.g. data owners or the queriers) are not involved in processing genomic data except for the result’s decryption. As discussed, involving such parties only during the decryption (or partial decryption) provides inherent access control for the proposed framework.

Additionally, (iii) providing interoperability between various genomic data uses improves the proposed scheme’s security and adoption. Security improves since data owners are not required to decrypt and re-encrypt their data for different genomic tests. Also, the adoption of the proposed scheme improves since data owners are allowed to use their data in various ways under the same system settings.

The proposed framework’s interoperability enables data owners to process their genomes individually, perform tests with the other data owners, analyze genomic databases, or conduct operations involving multiple databases under the same system model. Also, in the proposed framework, individuals’ genomic data are encrypted and stored at the SPU once during the initiation of the protocol. Thus, individuals can conduct (or request) multiple tests for healthcare purposes or use DTC services using this stored genomic information. Similarly, the hospitals encrypt their databases only once. Then, they can process these stored data to perform all four tests mentioned earlier. Besides, (iv) the capability of processing genomic data from different sources or data owners that are confidentially stored under each data owner’s unique cryptographic key all at once increases the scheme’s efficiency and practicality. As discussed, by delivering features (i)–(iv) simultaneously, the proposed framework is a promising protocol for interoperable and multiparty genomic data processing.

## 8 Conclusion and future work

Our paper introduces a cutting-edge privacy-preserving framework for genomic computations using mKH encryption. Unlike existing solutions, our approach allows data to be encrypted once and used for multiple analyses without re-encryption or additional setup. The distributed key management in our framework eliminates single points of failure, and our batching technique ensures stable performance across input sizes. While our solution may entail additional computational overhead, the trade-off is justified by the enhanced privacy guarantees, reduced communication rounds, and superior flexibility in handling diverse genomic tests. Although unsuitable for large-scale genomic studies, our framework offers a valuable alternative for applications where leaving encrypted data for multiple potential analyses is advantageous. Our future work will focus on optimizing performance for larger datasets and addressing potential inference attacks, further enhancing the utility of our approach in the rapidly evolving field of genomic data analyses.

Conflict of interest: Carmela Troncoso, Alptekin Kupcu, Juan Ramon Trocoso Pastoriza have declared conflicts of interest.

## Funding

We acknowledge that this work is partly funded by the Spanish Ministry for Science and Innovation and NextGenerationEU/PRTR through project “FELDSPAR: Federated Learning with Model Ownership Protection and Privacy Armoring” [MCIN/AEI/10.13039/501100011033], and by Xunta de Galicia and the European Regional Development Fund, under project ED431C 2021/47. Moreover, the first author acknowledges funding from the PID2021-125962OB-C31 “SECURING” project funded by the Ministerio de Ciencia e Innovación, la Agencia Estatal de Investigación and the European Regional Development Fund (ERDF), as well as the ARTEMISA International Chair of Cybersecurity and the DANGER Strategic Project of Cybersecurity, both funded by the Spanish National Institute of Cybersecurity through the European Union – NextGenerationEU and the Recovery, Transformation and Resilience Plan. Finally, the work is partly supported by the National Library of Medicine of the National Institutes of Health under Award Numbers R01LM013429 and R01LM014520, and by the National Science Foundation (NSF) under grant numbers 2141622, 2050410, 2427505, and OAC-2112606.

## Author Contributions

Mohammadali Farahpoor (Visualization [equal])
